# Microglia: Immune Regulators of Neurodevelopment

**DOI:** 10.3389/fimmu.2018.02576

**Published:** 2018-11-07

**Authors:** Maureen Cowan, William A. Petri

**Affiliations:** ^1^Department of Neuroscience, University of Virginia, Charlottesville, VA, United States; ^2^Division of Infectious Diseases and International Health, Department of Medicine, University of Virginia, Charlottesville, VA, United States

**Keywords:** microglia, maternal immune activation, synaptic pruning, microbiome, neurodevelopement, gut-brain-axis

## Abstract

Microglia, the tissue-resident macrophages of the central nervous system (CNS), have characterized roles in combating infection, clearing cellular debris, and maintaining tissue homeostasis. In addition to these typical immunological roles, microglia have been revealed to be active players in complex neurodevelopmental programs such as neurogenesis and synaptic pruning, during which they interact with neurons and macroglia to provide trophic support, respond to cytokine, and metabolic signals derived from the local neural environment, and drive the refinement of functional neuronal circuits. Microglia appear to be developmentally regulated by the host microbiome, and have been shown to dynamically respond to metabolic products from gut microbiota in a sex-dependent manner. Due to their constant surveillance of the brain parenchyma, involvement in development, and salient reactivity to both peripheral immune and microbiome-derived signals, microglia may additionally serve as a key cellular intermediate linking neurodevelopmental disorders such as autism and schizophrenia with microbiota influences in models of maternal immune activation (MIA). This review examines both well-established and emerging literature and perspectives on microglia in the context of neurodevelopment, with a particular emphasis on the role of the host microbiome in influencing microglial function during health and disease states.

## Microglia are brain-resident macrophages of unique origin and function

Under steady-state conditions, microglia serve as the sole immune cells of the central nervous system (CNS). As macrophages, these myeloid cells have been implicated in a wide array of CNS processes such as mediating inflammation, directly combating infection, and clearing cellular debris via phagocytosis [reviewed in (1)]. In addition to these “traditional” macrophage-type roles, microglia have more recently been revealed to be critical players in complex neurodevelopmental programs, during which their phagocytic and signaling repertoires have been shown to: (1) determine neuronal fate by regulating programmed cell death (2), (2) promote neurite formation, synaptogenesis, and axonal fasciculation (3), and (3) strip excess synapses from developing neurons to permit the assembly of functional neuronal circuits (4, 5). While some features of closely regulated pro-inflammatory activity are necessary for healthy neurodevelopment, unrestrained early-life inflammation may alter programming of the microglial population itself, leading for a lower baseline required for reactivation, thereby perpetuating inflammatory damage to the neuronal compartment later in life (6).

Microglia possess a unique origin that grants them a specialized niche as the tissue-resident macrophages of what has traditionally been viewed as an immune-privileged site. Fate-mapping studies have revealed that microglia arise almost entirely from yolk-sac erythromyeloid progenitors that seed the developing neuroepithelium and establish residency in the CNS (7). This cell population is segregated from peripheral hematopoietic contribution upon formation of the blood-brain-barrier (BBB) around E13.5, and self-renews throughout the lifespan (8). At steady state, microglia express a TGF- β driven transcriptional signature, expressing genes such as *Tmem119, Sall1, Tgfbr1*, and *P2ry12*, which distinguish them from bone marrow-derived macrophages (BMDMs) (9). Even after engrafting into the brain parenchyma BMDMs fail to acquire a microglial transcriptional profile, despite previous notions of macrophages being transcriptionally plastic and largely informed by their microenvironments (10, 11). The microglia-specific gene signature, which has been proposed to be regulated by the transcription factor *Sall1*, is thought to contribute to the cell population's relatively quiescent phenotype as compared to bone-marrow-derived macrophages in the unique CNS environment (12).

While necessary for certain aspects of CNS development and homeostasis, microglia can be targeted and depleted using multiple approaches to elucidate their functions. For instance, PU.1 is a transcription factor necessary for the development and viability of microglia (13). While PU.1-deficient mice die shortly after birth, adequate PU.1 levels in microglia are required for proper phagocytic capacity. Microglia in mice have been depleted via the expression of diphtheria-toxin under the CX3CR1 promoter, and antagonism of the CSF-1 receptor, which is expressed by microglia (as well as other macrophages) and also required for microglial viability (14, 15). Because microglia comprise a self-renewing population, inducible cre systems additionally allow for the selective genetic targeting of long-lived CX3CR1-expressing macrophages under various homeostatic conditions, during microglia are the only resident macrophage population in the CNS (16, 17). The relative ease of manipulating microglia makes them an attractive target for better understanding the etiology of neurodevelopmental disorders.

## Early-life inflammation and potential roles for microglia in immune-driven animal models of autism and schizophrenia

Microglia are thought to play an essential role in the process of neurogenesis, or the differentiation and maturation of precursors into neurons. (18). A majority of newborn neurons undergo apoptosis and are phagocytosed by microglia as part of normal neurodevelopment, and over time, this process becomes limited into neurogenic niches of the adult brain (19). Microglia not only play a critical role not only in debris clearance necessary to account for the number of apoptotic cells present resulting from neurogenesis, but may also instruct neuroblast differentiation in response to signals such as IFN-γ (20). However, transgenic overexpression of the pro-inflammatory cytokine, IL-6 results in a substantial decrease in neurogenesis in the absence of neuronal death, with concomitantly observed microgliosis (21). These features may contribute to developmental deficits stemming from early-life infection and inflammation, where sensitive neurodevelopmental programs are disrupted, as has been suggested in neurological disorders such as autism spectrum disorders (ASD) and schizophrenia. Studies investigating the role of microglia in neurogenesis typically use *in vitro* approaches or examine adult neurogenesis, due to relative challenges in studying embryonic systems. Nonetheless, they provide insights that collectively present contributions by microglia to fundamental aspects of neurogenesis through phagocytic and signaling activity, and their ability to respond to and potentiate inflammation within the CNS.

Autism spectrum disorder (ASD) and schizophrenia are neurological diseases that display a male sex bias that has been well described in meta-analyses over the past few decades (22, 23). The symptoms of these disorders can be recapitulated in the laboratory with rodents—both mice and rats. Parameters for symptomology in animal subjects typically evaluate behaviors typically affected in ASD or schizophrenic individuals—ultrasonic vocalizations (language), social interactions, repetitive behaviors, as well as anxiety phenotypes, using a battery of behavioral tests (24). Maternal immune activation (MIA) in rodents has become widely used to produce and study these phenotypes, as infection during pregnancy has been linked to both ASD and schizophrenia. Interestingly, the MIA model produces litters of mice displaying a distinct sex bias that mirrors that of ASD and schizophrenia–males are more likely symptomatic, whereas females are typically unaffected. Recently, a strong link between maternal Th_17_-derived IL-17 and the MIA phenotype in offspring has been established (25). However, microglia, due to their salient reactivity to the microbiome and roles in neurodevelopment, may additionally serve as an attractive downstream intermediate for contributing to the pathology observed in MIA at the level of the CNS.

An early paper from Paul Patterson's group prominently illustrated that mice born to dams that experience an immune challenge during gestation, namely human influenza infection, exhibited marked behavioral impairments including: (1) decreased exploratory (open-field maze), (2) reduced social activity (novel mouse test), and (3) deficits in pre-pulse inhibition that were alleviated by treatment with antipsychotic drugs (26). This paper further demonstrated that injection of the synthetic dsRNA viral mimic, polyriboinosinic-polyribocytidilic acid (poly I:C), was sufficient to recapitulate the behavioral deficits observed in MIA offspring. Subsequently, the pro-inflammatory cytokine IL-6 has been revealed as an intermediary in establishing neurodevelopmental deficits in offspring, as IL-6 administration alone is sufficient to cause pathology, and IL-6 neutralizing antibodies are able to rescue the MIA phenotype (27).

Recently, maternal immune activation in mice has been shown to result in an altered microglial transcriptional profile, characterized by changes such as decreased expression of phagocytosis-associated genes and increased cell motility–changes that (1) in part, parallel the neurodegenerative APPPS1-21 model for Alzheimer's Disease, and (2) were reversed with minocycline treatment (28). Some evidence suggests that, minocycline, an antibiotic drug that exerts a suppressive effect on microglial activation, may have therapeutic benefits in schizophrenic patients. (29, 30). One may speculate that disturbances to the microglial transcriptome, particularly decreased phagocytosis, could compromise synaptic refinement or roles supporting neurogenesis—leading to aberrant synaptic connectivity or changes to the survival fate of migrating neuroblasts. Alternatively, minocycline in this model could be exerting its effect directly on neurons, as neuronal patch-clamp, calcium imaging, and behavioral studies have shown it to (1) suppress excitatory glutamatergic transmission in rat hippocampal neurons, and (2) modulate rat behavior in forced swimming/open-field tests as models for depression in a MIA-independent context (31, 32).

In addition to difficulty in parsing out whether microglia are the causative agents in MIA behavioral phenotypes, this field is challenged by some major caveats to using MIA to represent human disease. While MIA reliably produces some behavioral phenotypes and distinct sex-bias in mice that are analogous to patients with autism or schizophrenia, it is limited as a surrogate for human disorders. Autism and schizophrenia are distinct conditions–one developmental, the other psychiatric–and they manifest with differential diagnostic criteria that are not parametrically compatible with existing rodent behavioral tests. The diagnostic criteria for schizophrenia, for instance, include hallucinations, delusions, disorganized speech, and catatonia (33). Some features of the hallmarks of autism (social and communication deficits, and repetitive behavior) are displayed in MIA mice, but it is similarly difficult to generalize them to complex neurological disorders. While challenging, and despite these flaws, MIA remains a valuable tool in beginning to understand an immune-based etiology for neurodevelopmental disorders, until the development of better-suited developmental neuroimmunological models.

## Microglia play critical roles in synaptic refinement of the developing CNS

By temporarily suturing shut the eyes of kittens, Nobel Laureates David Hubel, and Torsten Wiesel found that early-life visual binocular stimulation was required for neurons to establish normal electrophysiological responses in the striate cortex, and to establish typical cytoarchitecture in the visual thalamus (34). In a series of studies, they were amongst the first to develop the notion of “critical periods” of neural development, or designated biological time frames during which specific neural circuits or developmental phenomena are established. Early in life, the brain is highly plastic and shaped by sensory experience. An excess number of immature synaptic connections between neurons is initially established in the brain, and subsequently “pruned,” or eliminated, to establish functional connectivity during development. Decades after Hubel and Wiesel, glial biologists have identified microglia to be key developmental players during critical periods of synaptic pruning in brain regions including the retinogeniculate system (5, 35) and the hippocampus (4).

**Figure 1:**
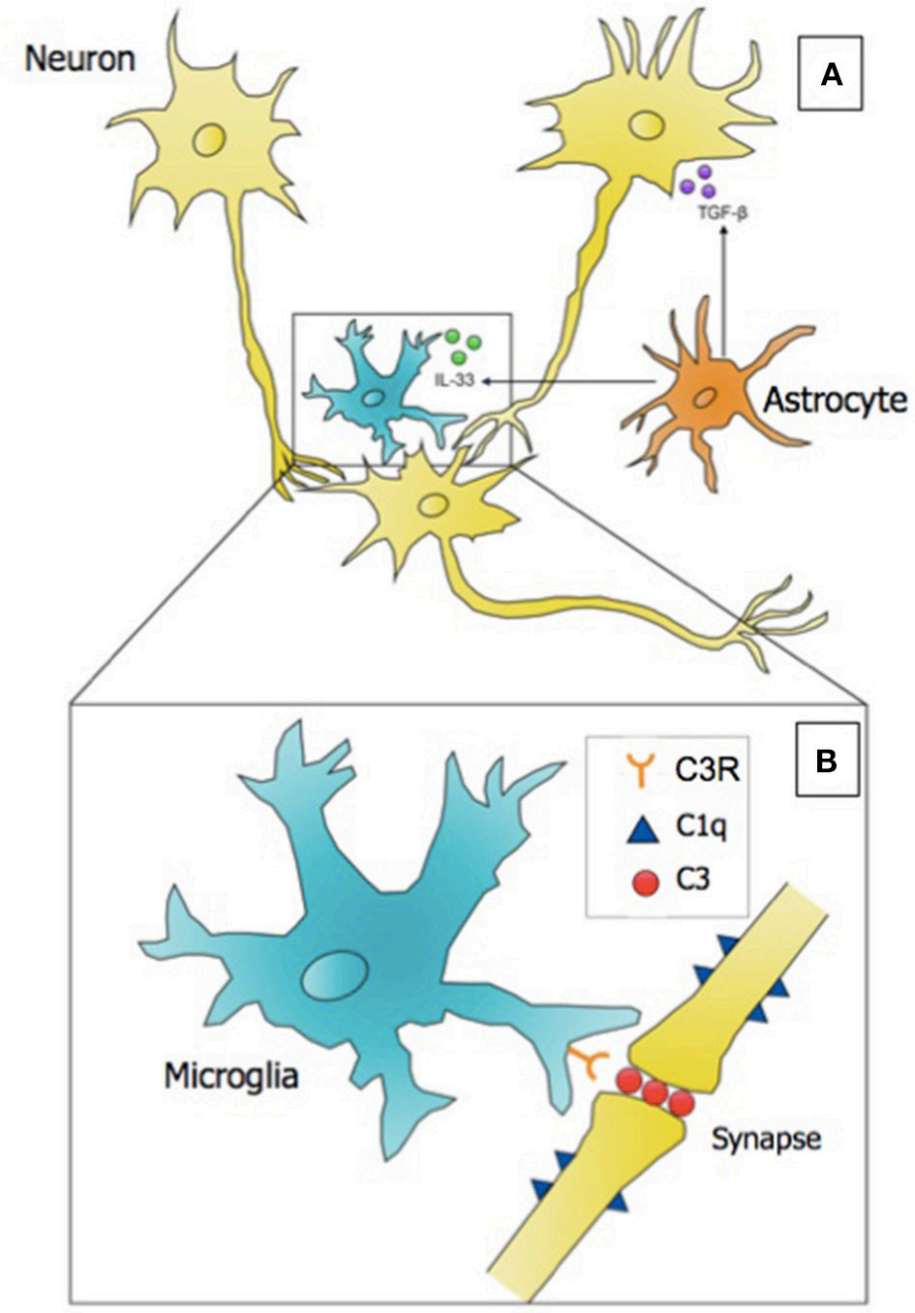
Microglia, astrocytes, and neurons interact to successfully eliminate weak or nonfunctional synaptic connections in the developing brain in the process referred to as synaptic pruning. **(A)** A microglial cell is depicted targeting a weaker synaptic connection. Astrocytes secrete two key cytokines: (1) TGF-β, which induces the expression of the initiating classical complement cascade protein (C1q) on neurons, and (2) the alarmin IL-33, which promotes the phagocytosis of synapses by microglia. **(B)** Inset of A; at the site of a weak synapse, neurons express complement component 1q (C1q), which serves as an “eat-me” signal to microglia. Microglia opsonize the synapse with complement component 3 (C3), and perform receptor-mediated phagocytosis using the C3 Receptor (C3R) to eliminate the synapse. Evidence suggests that synaptic pruning is regulated by the microbiome, and deficits in neural connectivity are observed in neurodevelopmental disorders including autism and schizophrenia. Punishment model adapted from Stephan et al. (37).

As tissue-resident macrophages, microglia are the key cell population in the CNS expressing complement protein 3 (C3) and its receptor C3R. In the developing visual system of the mouse, they have been shown to opsonize and engulf competing synaptic elements in developing neuronal circuits (5, 35). This process has been proposed to be activity dependent, as pharmacologically inhibiting or promoting retinal ganglion cell (RGC) activity led to alterations in synaptic terminal field size in the dorsal lateral geniculate nucleus (dLGN), the visual thalamus. Astrocyte-derived TGF-β was subsequently identified as the initiating cytokine signal to initiate neuronal C1q expression, activating the classical complement cascade and uptake of synaptic components by microglia via C3R (36). Labeling RGCs with fluorophore-conjugated cholera toxin subunit B (CTB), which travels in an anterograde fashion to axon terminals, revealed labeled synaptic content localized within the microglial phagolysosome. The model that has emerged from microglial studies in the retinogeniculate system proposes a “punishment model,” whereby neuronal synapses that are weaker, or less functional, fail to establish a protective molecular signal that prevents their engulfment by microglia, phagocytes tasked with stripping away weaker synapses in order to sculpt functional synaptic circuits (37). Further components of this synaptic pruning model have been expanded since it's conceptualization, as depicted in Figure 1. While this model is intriguing, there remains some skepticism as to whether microglia are surgically excising opsonized synapses, or clearing synaptic debris that was eliminated by some other mechanism, such as neuronal shedding (38).

Recent developments in the story of microglial synaptic pruning have also implicated a role for microglia in responding to the type-2 alarmin, interleukin-33 (IL-33), derived from astrocytes, to promote synaptic pruning in the reticular thalamic nucleus, as well as the hippocampus (39). IL-33 knockout mice were shown to display deficits in synaptic elimination during development, as evidenced by decreased post-synaptic density 95 (PSD95)-labeled synaptic puncta localized within GFP-expressing microglia, impaired sensorimotor (startle) responses, and increased excitatory post-synaptic potentials from excited neurons indicative of overabundant presynaptic inputs to recorded neurons. In addition to IL-33, neuronal CX3CR1 (fractalkine) signaling has been shown to serve as a spatiotemporal regulator of microglial number and pruning activity in the hippocampus (4), but does not appear to play a universal developmental role in other areas of the brain, such as the visual cortex (40). Because macrophages are functionally informed by environmental cues and different regions of the CNS possess unique molecular environments, microglia may be viewed as a heterogeneous population *in vivo*, and a one-size-fits all approach for understanding glial-glial or neuronal-glial signaling appears limited. Indeed, microglial morphological complexity, motility, electrophysiological properties, and transcriptional properties vary in a location-dependent manner (41).

Deficits in synaptic pruning are thought to play a role in conditions such as ASD and schizophrenia, where either hyperconnectivity and/or hypoconnectivity is observed across regions including the amygdala, pre-frontal cortex, and components of the default mode network (42–44). A recent study has suggested a requirement for the phagocytic receptor TREM2, expressed by microglia in the CNS, in regulating phagocytic capacity needed to drive synaptic elimination in subregions of the hippocampus (45). *Trem2*^−/−^ mice show an increased expression of synaptic proteins such as PSD95 and Shank2, an increased number of dendritic spines, increased functional connectivity between multiple brain regions as evaluated with fMRI, and increased miniature excitatory post-synaptic currents (mEPSCs) in neuronal patch-clamp recordings. These molecular, neuroimaging, and electrophysiological changes in *Trem2*^−/−^ mice are suggestive of hyperconnectivity and hyper-excitability of circuits, converging on observed social and repetitive behaviors that parallel those of ASD symptoms. These findings suggest that deficits in microglial phagocytic function can perturb neurodevelopment. A more recent paper has suggested that microglia may play a role in dopaminergic circuit refinement via CR3-mediated phagocytosis (46). Pharmacologically injecting a CR3 subunit antagonist into the nucleus accumbens, which blocks the ability of local microglia to phagocytose complement-tagged material, resulted in deficits to D1R (Dopamine Receptor D1) elimination during defined critical periods in rats. Neuronal dopaminergic signaling is critical in reward-seeking and social behavior, and compromising microglial clearance of D1Rs resulted in decreased levels of social play and social exploration in adolescent male rats, thus accumulating evidence for behavioral outputs for deficient synaptic refinement by microglia.

## The gut-brain axis: the gut microbiome influences neural and microglial activity

The microbiome has been linked to the CNS via different proposed communication pathways: (1) bacterial metabolic products such as tryptophan and short chain fatty acids (SCFA), (2) innervation of the gut by the vagus nerve, and (3) microbe-associated molecular patterns (MAMPs) that drive inflammation [reviewed in (47)]. The mechanism by which bacterial products or MAMPs trigger an inflammatory response in the brain may include entry to the circumventricular organs, areas in the brain where the BBB is less tightly regulated (48). While these molecules are limited in their entry throughout the BBB, cells present at these “leaky” locations (i.e., microglia), may be poised to respond to blood-borne molecular signals, and in turn propagate inflammation in a wave across the parenchyma. Alternatively, microglia may respond to cytokine-induced reactivation of cells forming the BBB–endothelial cells, astrocytes, pericytes, etc; or via selective transport systems across these cells (49–51). A neural route for the gut-brain axis suggests that a basal level of immune activation in the gut acts on the vagal afferents innervating the viscera and transduces inflammatory information to the nucleus of the solitary tract (NTS), at the level of the brainstem (48, 52). Low levels of cytokines may activate this route, even when circulating pro-inflammatory cytokines are not detectable (53). This vagal-centric model for understanding gut-brain-axis has not yet been approached from the angle of microglial development, activation, or function, and remains of interest to the field.

Whether through metabolic, humoral, or neural routes, microglia display highly motile branching processes that extend and sample the entire brain parenchyma, a phenomenon that has been widely described as a surveillance function (54). Microglia express a complex sensome, and are capable of integrating and responding to signals in their environment, whether PAMPs, DAMPs, or other secreted factors, by secreting pro-inflammatory cytokines including TNF- α, IL-1 β, and IL-6 (55, 56). These signaling features, their macrophage identity, and status as resident-CNS cells make microglia intriguing candidates for mediating the interactions between the host's microbiome and developing brain. Indeed, metabolic products from gut microbiota, as well as microbiota-induced peripheral immune products are capable of influencing microglial activity.

An extensive investigation of microglia as intermediates of the microbiome and the developing brain was recently performed—using RNA- and ATAC-seq analyses of germ-free vs. SPF mouse embryos (57). Microglia were found to adhere to a generalized developmental trajectory upon colonizing the brain, with distinct transcriptional profiles that differed between males and females, as has also been characterized in a new microglial “developmental index” in the context of LPS-induced inflammation (58). Interestingly, the effects of germ-free conditions were both age and sex-dependent, with male germ-free mice displaying more differentially expressed genes relative to females during embryonic development, but fewer during adulthood. The microbiome additionally influenced patterns of microglial chromatin accessibility, as evaluated via ATAC-seq, in a sex-dependent manner. These experiments add to the rapidly growing field of microbiome-CNS interactions in the context of evaluating microglia under a neurodevelopmental lens. They additionally contribute to an emerging understanding of sex differences in understanding microglial development and function, which is of particular interest in studying neurological disorders displaying a sex-bias.

There are examples of gut microbiome metabolic products modulating the peripheral immune system, notably short chain fatty acids such as butyrate that affect T regulatory cell development (59). However, action on the CNS of microbial metabolic products from the gut microbiome is a new observation. In terms of direct effects of the microbiome on microglial activity, these observations are of new interest, but still limited in number. One study has shown that microglia isolated from germ-free mice display a range of morphological, molecular, and spatial abnormalities–increased density across various brain regions, altered cytometric expression patterns for developmentally regulated proteins such as CSF1R, CD31, and the activation marker F4/80, exaggerated process lengths, and disrupted distribution across the brain (60). Of note, the observed protein expression differences observed in microglia derived from germ-free mice correspond to developmental profiles of immature microglia. These microglial changes were shown to be dependent on SCFA, as SPF mice constitutively lacking the SCFA receptor FFAR2 displayed a similar aberrant phenotype to germ-free animals. In these same experiments, germ-free and antibiotic-treated conditions were shown to upset the typical microglial spatial network throughout the brain, with microglia failing to evenly tile, and forming atypical contacts between processes of adjacent cells. The ability of microglia to tile across the brain with discrete territories is an interesting feature of these cells, but the field has yet to provide an explanation for why tiling may become disrupted, or what deficits in tiling may functionally represent. Microglia from GF mice in this study were additionally found to be less reactive when challenged with LPS relative to controls, which raises the question as to whether an anti-inflammatory immune response may be, in this case, neuroprotective.

The findings from the Erny paper suggest a role for the microbiome actively informing microglial development and homeostasis, but a few caveats exist. Optimal colonocyte metabolism depends on the oxidation of microbiota-derived butyrate, a SCFA (61). In the absence of butyrate, it is conceivable that inefficient energy metabolism at the level of the gut, may shift metabolic profiles systemically and in the brain, irrespective of microglial activity. More notably, brain-resident cells, including microglia, were not shown to express detectible levels of FFAR2 mRNA. An alternative explanation may propose that peripheral cells expressing FFAR2 transduce a cytokine (“humoral”) signal that is then propagated within the brain by microglia. Still, how SCFA restores microglial disruptions remains unexplored.

As with any experimental system, it is important to note that the use of germ-free mice and administration of antibiotic cocktails present their limitations to cleanly dissecting observed results implicating a direct microglial response to gut microbiota. GF display impaired immunological development, with a phenotype characterized by decreased numbers of specific T cell subsets, stunted intestinal and mesenteric lymph node development, deficient IgA production, and increased susceptibility to infections including *Shigella* and *Listeria* [reviewed in (62)]. It is also difficult to predict the generalizability of findings from studies involving GF mice to the context of human disease. Besides standard points that mice are not humans, and humans do not live in GF conditions, there is considerable variability in the behavioral and cognitive phenotypes observed in GF mice of different strains and sexes, as has been exposed with a battery of behavioral tests across many studies [reviewed in (63)].

More recently, tryptophan metabolites have now been demonstrated to act directly on microglia in the brain (64). This link of gut microbiome to CNS microglia was originally suggested by the fact that genetic knockout of the aryl hydrocarbon receptor (AHR) affected the progression of experimental autoimmune encephalomyelitis (EAE) in the mouse model (65, 66). At the time this was thought to be due to the role of AHR in Treg and Th17 cell differentiation in the periphery. More recently came the understanding that AHR might be acting directly in the CNS. AHR was discovered to be expressed not only in T cells in the periphery but also by microglia and astrocytes in the brain (67). Activation of AHR by the bacterial metabolites of tryptophan (indole, indoxyl-3-sulfate, indole-3-propionic acid and indole-3-aldehyde) was shown to attenuate inflammation in EAE. Conversely, conditional knockout of AHR in microglia resulted in increased inflammation in EAE—results consistent with direct activation of AHR via bacterial products. That these tryptophan metabolites were the product of gut bacteria was demonstrated in part by antibiotic treatment of the mice increasing inflammation, an effect that could be overcome by addition of bacterial tryptophanase and by diets rich in tryptophan. AHR activation led to induction of TGF-α and repression of VEGF-β that in turn acted on astrocytes to decrease CNS inflammation (64).

## Concluding remarks

Microglia possess a myriad of properties that make them an attractive candidate effector mediating microbiome-brain interactions. As professional resident phagocytes, they maintain a poised position in the CNS, possess a wide repertoire of signaling contributions to developmental programs, and display dynamic morphological and transcriptional responses to gut microbiota. Rather than simply being macrophages in the brain, there has additionally been a shift to regarding microglia as a specific population of CNS-resident cells that are distinctly informed by not only their environment, but also ontogeny. This ontogeny is important to consider in the case of the developing brain, where exceedingly complex neuronal architecture must be established within defined critical periods, with minimal inflammatory insults or perturbations.

Experiments examining the role of microglia in developmental synaptic pruning may encompass a large portion of our understanding of these cells, as microglia seem to be required the normal refinement of synaptic circuits, but it is currently unknown as to whether they are actively removing specific synapses with surgical precision, or passively phagocytosing them after they have been removed via other means. Moreover, a microglia-centric view of synaptic pruning in the developing brain, while initially exciting to the field, appears incomplete. Astrocytes, which outnumber microglia up to 10-fold during the critical period of dLGN pruning, have been shown to engulf a greater cumulative amount of CTB-labeled synapses than microglia (although with lower per-cell efficiency), in a MERTK and MEGF10-dependent manner (68). Whether early-life inflammation affects astrocytes in a way that compromises their complement-independent pruning programs may be of interest. The field of developmental neuroimmunology may similarly benefit from experiments showing that germ free conditions affect known microglial-dependent pruning processes, especially during currently defined critical periods of development that may lead to consistent behavioral phenotypes.

The results that SCFAs and tryptophan metabolites from microbiota may act directly on microglia may have direct applicability to the pervasive problem of neurocognitive delay in infants growing up in adversity in low income countries. The enormous burden of enteric infections in these children results in gut microbiome dysbiosis (69), malnutrition and chronic inflammation (70), all of which are linked in turn to neurocognitive deficits as measured by tests of infant development (71, 72). The work of Rothhammer et al. discussed earlier, suggests one mechanism that this may occur: we and others have discovered that tryptophan is deficient in the plasma of malnourished children. In light of their studies on tryptophan metabolites influencing microglial-driven inflammation, Rothhammer et al would predict that this deficiency of tryptophan would lead to chronic CNS inflammation via AHR-mediated reduction in microglia TGF-α. The encouraging aspect is that the CNS damage suffered by children living in adversity might in part be amenable to probiotic or dietary therapy.

Overall, a growing field of evidence describes (1) associations between microglia and neurodevelopmental health and disease, (2) a potential functional link between microglial activity and the host microbiome, and (3) influences by the microbiome on CNS development and animal behavior. To date, the critical missing component in this microglial-microbiome-development story is robust evidence that disruption of the microbiome compromises microglial developmental programs such as neurogenesis and synaptic pruning, in a way that drives neurodevelopmental phenotypes such as ASD or schizophrenia.

## Author contributions

WP: Conceptualization of the manuscript, reviewing and editing the manuscript. MC: Writing the manuscript.

### Conflict of interest statement

The authors declare that the research was conducted in the absence of any commercial or financial relationships that could be construed as a potential conflict of interest.
